# Commentary: Chasing the Rainbow: The Non-conscious Nature of Being

**DOI:** 10.3389/fpsyg.2018.00372

**Published:** 2018-03-22

**Authors:** Philip Clapson

**Affiliations:** Centre for Brain and Cognitive Development, Birkbeck University of London, London, United Kingdom

**Keywords:** brain-sign, consciousness, neural communication, organismic communication, collective action

The authors of this paper (Oakley and Halligan, 2017) have an ambitious theory: Alter the customary view that consciousness has a causal role in the brain's activity, and the functioning of the organism. They propose that “all psychological processing and psychological products are the products of fast efficient non-conscious systems.” What has been supposed as the defining characteristic of particularly human life, consciousness, “is a passive accompaniment to the non-conscious processes.” This, they say, is a “logical conclusion” from a growing consensus over the last 30 years.

They acknowledge that epiphenomenalism is not a new idea, and that non-conscious activity is generally accepted. In fact Lancelot Law Whyte proposed in 1960 (see Whyte, 1960) that “everything that man does and thinks arises from this challenging phenomenon: *the unconscious genesis of mental processes*.” He dated the beginnings of the idea to 1700. The twist the authors offer is that these non-conscious processes generate the Personal narrative (PN), which “has compelling real-world face validity—particularly when linked to the notions of ‘self’ and ‘personhood.’ While previously attributed to ‘consciousness’—given its temporal association with the same—in our account PN is not produced or in any way constrained by conscious experience” and crucially this personal narrative serves “External and Cultural Broadcasting [which] are supra-individual.” This is a mechanism for interbrain communication.

The difficulty the authors face is explaining how and why that with which we are completely familiar has no causal power, but does exist in a way that is invisible to us as consciousness. Indeed they say that they “have no option” but to use psychological language to describe this personal narrative because they do not reform it in any way. So we have what seems an extraordinary situation in which we know there are psychological states because we are conscious, but our knowledge is irrelevant because what we know has no causal authority. Indeed, they recognize this. “The exquisite temporal contiguity between personal awareness [consciousness] and the contents of the personal narrative have … provided a reliable … explanation for a compelling causal association between the two that remains particularly difficult to argue against.”

Actually, Oakley and Halligan cannot hold to their theory. For example, they say that “‘Personal awareness’ [consciousness] just ‘is’, though as humans we feel compelled to ‘explain’ it by attributing a functional capacity, purpose, or meaning to it, and in so doing, we argue, has generated a host of misconceptions.”

This passage exposes not merely the difficulty of arguing for non-conscious psychological states, but avoiding causal consciousness itself. The argument employs us “as humans” rather than brain functioning, and these humans feel compelled to “explain” by “attributing a functional capacity,” etc. Unless they are proposing there can be non-conscious feeling, then consciousness has emerged as an active agent in brain functioning. This consciousness leak undermines the argument generally.

Of course, their argument might be that feeling can be non-conscious, presumably like seeing and sensing! But the problem here is the analogy of the rainbow to Thomas Huxley's epiphenomenal steam whistle. The whistle does nothing causal; neither does the rainbow. Unfortunately, as with the consciousness leak, the argument fails, for rainbows occur to viewers, and no one views consciousness. Consciousness (e.g., feeling, seeing and sensing) just “is,” as they say.

Which brings us to the crux of the difficulty. The foundational terms appear to designate things or states that exist as “a given.” This is explicitly said about personal awareness (consciousness), but equally applies to experience. If the aim is to construct a scientific account of brain functioning (which is mentioned on occasion), then all terms require to be given physical status *ab initio*. The authors simply avoid doing so, so one wonders what the aim of the paper is. Notoriously, the topic of consciousness possesses no scientific definitions, and millions of words are spilled with no defensible scientific outcome.

However, the authors do identify characteristics which require explanation. How can brains communicate with each other with purely physical properties? Their method is by making psychology non-conscious and rendering consciousness epiphenomenal. Neither of these is convincing. There is an alternative. That is given by the *theory of brain-sign* (model first published in 2001) which sets out to achieve precisely the same result, but by a different route.

Brain-sign theory states that consciousness does not exist, but there *is* an actual physical brain phenomenon, brain-sign. Brain-sign is the means/mechanism for interbrain communication in collective action. It is derived moment by moment from the causal orientation of each brain. Thus if A passes B a cup of coffee, each brain signifies the part of the world which is the focus of communal action, the cup of coffee (and much else besides). The brain generates the brain phenomenon, brain-sign, *from* the causal conditions and it has a biophysical role, which is to act as the pointer to the part of the world to which the causality of the brain is directed. Signification could not be by the actual causal conditions of the brain, for they are massively complex assemblies of electrochemical material. These causal conditions are, of course, not available ‘to us’ as brain-sign: but why would they be? ‘We’, as brain-sign, serve the brain's physical role in achieving survival and reproduction collectively. ‘We’ are not a knowledge characteristic of brains.

The theory is extensive, but can be accessed in published material (Clapson, 2014, 2016, 2017). However, a couple of contrasts can be made between it and the Oakley/Halligan account. Firstly a precise role is given to the brain phenomenon with its ontology that of a physical sign, an operational element across biology: for example, the chromatic changes of the chameleon or cuttlefish; the tail of the peacock; the call of the vervet monkey identifying specific predators. Secondly, brain-sign theory offers neuroscience both a coherent and precise way of approaching brain architecture, for brain-sign is part of brain function and not an unspecifiable entity operating in a different and conflicting way from the causal brain, i.e., consciousness. This is surely a more satisfactory option.

## Author contributions

The author confirms being the sole contributor of this work and approved it for publication.

### Conflict of interest statement

The author declares that the research was conducted in the absence of any commercial or financial relationships that could be construed as a potential conflict of interest.
